# Editorial: Bioactive Compounds With Potential Medicinal Properties Derived From Fungi: Recent and Future Developments in Microbial Biotechnology

**DOI:** 10.3389/fmicb.2022.837586

**Published:** 2022-02-01

**Authors:** Paola Angelini, Ahmed M. Abdel-Azeem, Carolina Elena Girometta

**Affiliations:** ^1^Department of Chemistry, Biology and Biotechnology, University of Perugia, Perugia, Italy; ^2^Department of Botany and Microbiology, Faculty of Science, Suez Canal University, Ismailia, Egypt; ^3^Department of Earth and Environmental Sciences (DSTA), University of Pavia, Pavia, Italy

**Keywords:** amazonian fungi, Ascomycota, Basidiomycota, biological activities, Chinese medicinal preparation, crude extract, marine fungi, secondary metabolites

The continuous exploration of new sources for drugs and bioactive compounds has never ceased to delve into the fungal kingdom. Indeed, it has entered profoundly into the widest range of environments on Earth as well as into the chemical structure of compounds and the molecular mechanisms underlying metabolism regulation.

It can be demonstrated once more that varying points of view within the scientific community can still be held whilst retaining consistency along several research paths, allowing the gradual convergence in obtaining solutions.

The work by Ding et al. is a perfectly serendipitous combination of molecular biology, organic chemistry, and plant pathology that starts from the transcription regulation to discover new compounds with antifungal activities. In this study we find a focus on polyketides, which is the most represented category of the secondary metabolism in Ascomycota unlikely terpenoids, whose deputed portion of the genome is significantly larger in Basidiomycota. However, Ascomycota have to date included the major model organisms in this concern, thus Shi et al. provide us with the discovery and characterization of six new tremulane sesquiterpenoids from *Pseudogymnoascus* sp. showing good potential against human cancer cell lines. Analogously, Ge et al. deal with new enantiomers of a NOR-bisabolane derivative and two new phthalides by *Penicillium chrysogenum* and suggest applications of these compounds against some important fungal phytopathogens. Further on, Pan et al. report four new indole-terpenoids with anti-inflammatory activities from *Penicillium* sp., and find a species from the poorly known Genus *Pseudogymnoascus* besides the model-species for biosynthetic pathways *P. chrysogenum*, to witness that new frontiers in mycology are to be conquered in both.

Several works in this issue share another common feature: their fungal sources come from the sea. “Marine” fungi are indeed related to different habitats. They can be isolated from the sea but actually their life is not actually dependent on this environment. However, in the sea they find peculiar niches which are likely to stimulate the adaptation of unknown strains against a range of bacteria including human pathogens. Thus, Wang et al. report a sponge-associated *Cymostachys* sp. from a marine mesophotic zone; the above mentioned Ge et al. have also achieved their strain from a marine environment as well as Jenssen et al., who also tested their strain against several human melanoma, hepatocellular carcinoma, and non-malignant lung fibroblast cell lines. This latter work introduces us to fungi which are to be regarded as more strictly related to the marine environments around the world. Further on, Pang et al. report the antibacterial activity of a *Penicillium* strain from tides.

In addition to the sea, the endophytic niche is another enormous and still widely unexplored source of fungal diversity and bioactive resources. Endophytes are difficult and sometimes ambiguous to define, creeping in a shadow zone among saprotrophism, mutualism, and potential parasitism—opportunism, basically. The endophytism topic meets the marine fungi topic when dealing with fungi hosted by algae, as Sahoo et al. have shown. Another topic that merits attention is the great potential of chitosan in biomaterials. Fungi are a major source of chitosan that is easily obtained from chitin; here, Li et al. describe the joint application of chitosan-based hydrogel and algal extracts to treat cutaneous wounds in mice without inducing excessive inflammatory response.

Returning to the endophytes, besides the mere search for new bioactive compounds, the study of fungal metabolites contributes to understanding the complex relationship between host and fungus as a stepping stone to improve the bioactivity itself, as reported by Charria-Girón et al. Moreover, this study highlights the importance of the valorization and preservation of the biodiversity in fragile habitats such as the tropical montane rainforest in Colombia.

The endophytism topic also entails the topic of the rhizosphere fungal community, including, according to Ding et al., genera like *Trichoderma*. Zhang et al. consider *Trichoderma* “a treasure house of structurally diverse secondary metabolites,” since it is an evergreen model for studies about fungal metabolite diversity. The plethora of bioactive compounds from *Trichoderma* species has current and potential applications in medicine and toxicology as well as in agriculture, phytopathology, and other fields.

The invisible role of fungi in the environmental equilibrium, even against invasive allochthonous parasites, is brought to our attention by Rodrigues de Oliveira et al. through their work on the larvicidal activities of fungal strains isolated from water against *Aedes aegypti* in the Amazonas. From outdoor water to controlled fermentation tanks for traditional or innovative products, this study reminds us that interspecific and intraspecific metabolic features produce a unique profile in the substrate. Simply due to the potential bioactivity of its component, such a profile must be handled very carefully. Thus, Xie et al. describe the evolution in the community participating in the fermentation process of the Chinese medicinal preparation of “Lianzhifan solution” as well as the evolution of its chemical profile. Notably, two species deserving particular attention are pointed out as key actors in the fermentation: *Aspergillus niger* and *Penicillium expansum*, which are typically double-edged swords due to their biotechnological as well as toxicological potential.

When dealing with bioactive compounds, the basic aims of the work may be multiple: research, separation, and structural characterization of new compounds; qualitative and quantitative exploration of the bioactivity on specific targets, without exact discrimination of the bioactive molecules acting on these; and both the matters together.

In this issue, four articles rely on the latter approach by either dealing with bacteria, virus, other competitor fungi, or animal parasites. For example, Grey et al. deal with the need of new antibiotics against *Mycobacteria*—also including *Mycobacterium tuberculosis* complex—by applying a screening of fungal species, even phylogenetically distant from each other, which may show antibacterial activity. In order to achieve this, the authors propose a medium-throughput bioluminescence-based assay, without regard to the exact identification of the molecule(s) responsible for the desired result, at least in this stage of the work. It is evident in such an approach that the fungal bioactivity is often the result of a wide range of compounds acting altogether. Above all, the challenge of fungal metabolites to analytics and structural chemistry is notable and full of technical difficulties. When they are not fully known, these compounds are often unknown to the libraries and their standards are really difficult to achieve. The same species can shift from certain metabolites to others depending on the growth stage and, of course, on the strain. The frontier is therefore still wide and open for scientists. In this issue, seven articles brought their contribution to the identification of new molecules. The readers will therefore find inspiration from the innovative combinations of methods for a multi-focus approach: HPLC and spectroscopic and spectrometric data furtherly supported by X-ray crystallography for Wang et al.; high resolution mass spectrometer (HRMS), NMR, and electronic circular dichroism (ECD) for Ding et al.; ECD spectra are the striking tool for Shi et al. as well as for Pan et al. and Pang et al. to confirm the potential of this technique; Ge et al. also adopt ECD besides 1D/2D NMR and ESI-MS; quite similarly, Jenssen et al. work by 1D/2D NMR and HRMS.

Last but not least, the challenge posed by the research and characterization of bioactive compounds cannot cast a shadow over another apparently minor topic that is far from being an aristocratic and sterile issue: the exact identification and taxonomic placement of the species and strains under examination. This is another story made of non-negligible endeavor and workload; no surprise if one cannot focus on both simultaneously. This expertise is often different from the one of bioactive compound explorers. This is not a limitation, this is just a confirmation of the African proverb: “If you want to go fast, go alone. If you want to go far, go together.”

## Author Contributions

PA first conceived and outlined the general topics of this special issue as well as the draft of the editorial. PA, CG, and AA-A contributed together to select and tailor the topics, to actively manage the papers as guest editors, that is by overseeing the special topic, to contribute to the editorial revision and final approval, and contributed to the writing of this editorial. All authors contributed to the article and approved the submitted version.

## Conflict of Interest

The authors declare that the research was conducted in the absence of any commercial or financial relationships that could be construed as a potential conflict of interest.

## Publisher's Note

All claims expressed in this article are solely those of the authors and do not necessarily represent those of their affiliated organizations, or those of the publisher, the editors and the reviewers. Any product that may be evaluated in this article, or claim that may be made by its manufacturer, is not guaranteed or endorsed by the publisher.

